# Secondary Diffuse Large B-cell Lymphoma Mimicking Meningioma

**DOI:** 10.7759/cureus.5833

**Published:** 2019-10-04

**Authors:** Miguel Garcia-Grimshaw, Diego Posadas-Pinto, Jesus Delgado-de la Mora, Amado Jimenez-Ruiz

**Affiliations:** 1 Neurology, Instituto Nacional de Ciencias Medicas y Nutricion Salvador Zubiran, Ciudad de Mexico, MEX; 2 Pathology, Instituto Nacional de Ciencias Medicas y Nutricion Salvador Zubiran, Ciudad de Mexico, MEX

**Keywords:** meningioma, lymphoma, brain neoplasm, diffuse large b cell lymphoma

## Abstract

Meningiomas are the most common benign intracranial tumors accounting for up to 30% of non-glial tumors of the central nervous system (CNS); on neuroimaging studies, they usually appear as a lobular, extra-axial mass with well-circumscribed margins mostly located in the parasagittal aspect of the cerebral convexity. On magnetic resonance imaging (MRI) of the brain, meningiomas are typically isointense to hypointense relative to grey matter in the T1-weighted sequence and isointense to slight hyperintense relative to grey matter on the T2-weighted sequence with avid homogeneous enhancement after contrast administration. A thin linear enhancement along the dura infiltrating away from the lesion, known as the dural tail sign, was once thought to be a pathognomonic feature of meningiomas, but this non-specific sign can also be seen in other meningioma-like lesions. Several benign and malignant pathologies may mimic some of the neuroimaging characteristics of meningiomas; among them dural metastases of lymphomas. When approaching a patient with suspected meningioma, close attention to the neuroimaging features may help distinguish them from meningioma-like lesions. Here we present the case of a woman with CNS involvement of non-Hodgkin lymphoma that presented with a dural mass resembling the neuroimaging characteristics of a meningioma.

## Introduction

Secondary central nervous system (CNS) involvement of aggressive systemic non-Hodgkin lymphomas occurs in less than 5% of diffuse large B-cell lymphomas (DLBCL) cases [1-2]. At the time of diagnosis, the most commonly affected site of the CNS is the brain parenchyma in up to 50% of the patients, followed by the meninges in 30%, and both sites in 16% of the cases. Despite the addition of rituximab as first-line therapy, the overall mortality remains as high as 80% during the first three months after initial diagnosis [1]. Gadolinium-enhanced magnetic resonance imaging (MRI) of the brain is the most sensitive neuroimaging technique for the detection of CNS lymphoma involvement [3]. A variety of conditions can mimic the clinical presentation and MRI patterns of CNS lymphoma among them meningiomas [4].

## Case presentation

**Figure 1:**
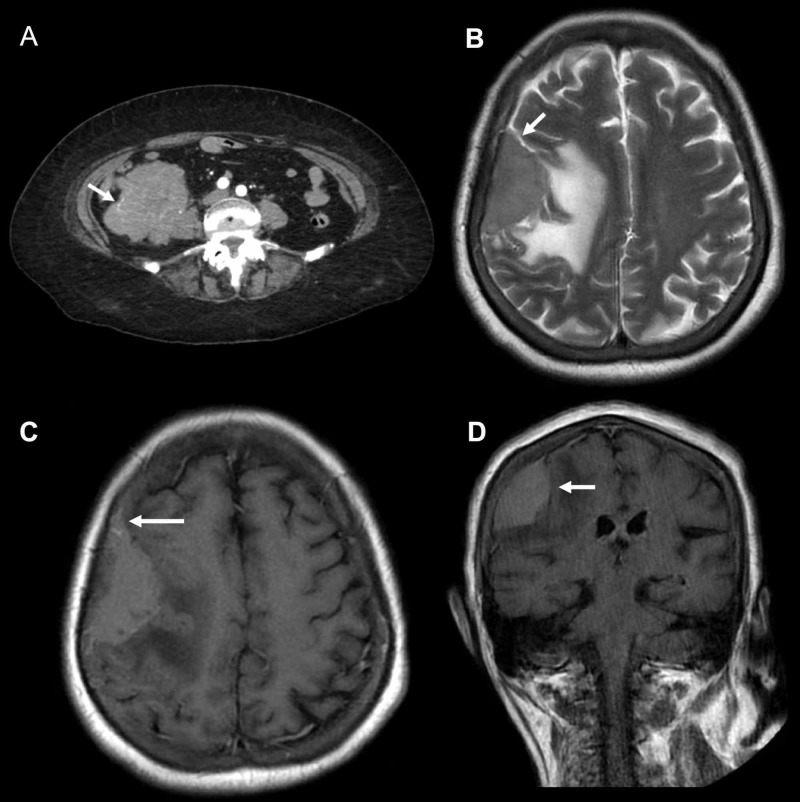
Abdominal computed tomography (CT) and brain magnetic resonance imaging (MRI) findings (A) Abdominal CT shows a contrast-enhancing ill-defined appendicular mass; (B) Axial T2-weighted brain MRI shows an extra-axial right frontotemporal dural mass with perilesional edema and a cerebrospinal fluid cleft (arrow); (C-D) Post-contrast axial and coronal T1-weighted MRI shows a heterogeneously enhancing round mass with well-circumscribed margins and dural tail sign.

A previously healthy 77-year-old woman, with a one-month history of headache and left arm weakness presented to the emergency department with nausea, vomiting, and abdominal pain for the past three days. On admission, she was alert; the neurological examination was relevant for left upper motor neuron facial paralysis and mild ipsilateral arm weakness (Medical Research Council (MRC) scale 4/5). An abdominal examination revealed a round palpable mass in the right lower quadrant. Blood workup, including a full blood count, serum electrolytes, lactate dehydrogenase, liver, and kidney function tests, were all within the normal range. Testing for human immunodeficiency virus (HIV) and hepatitis C antibodies were negative. An abdominal computed tomography (CT) showed a contrast-enhancing ill-defined appendicular mass (Figure 1A). Three days after presentation, while being prepared for a diagnostic colonoscopy, the patient developed two generalized tonic-clonic seizures; after the second episode, she persisted with a decreased level of consciousness for more than an hour requiring intubation.

As part of the seizure investigation, an electroencephalogram (EEG) was performed; relevant findings included an encephalopathic rhythm within the delta-theta range without epileptiform activity. MRI of the brain showed an extra-axial right frontotemporal dural mass with heterogeneous gadolinium enhancement and perilesional edema (Figures 1B-1D).

**Figure 2:**
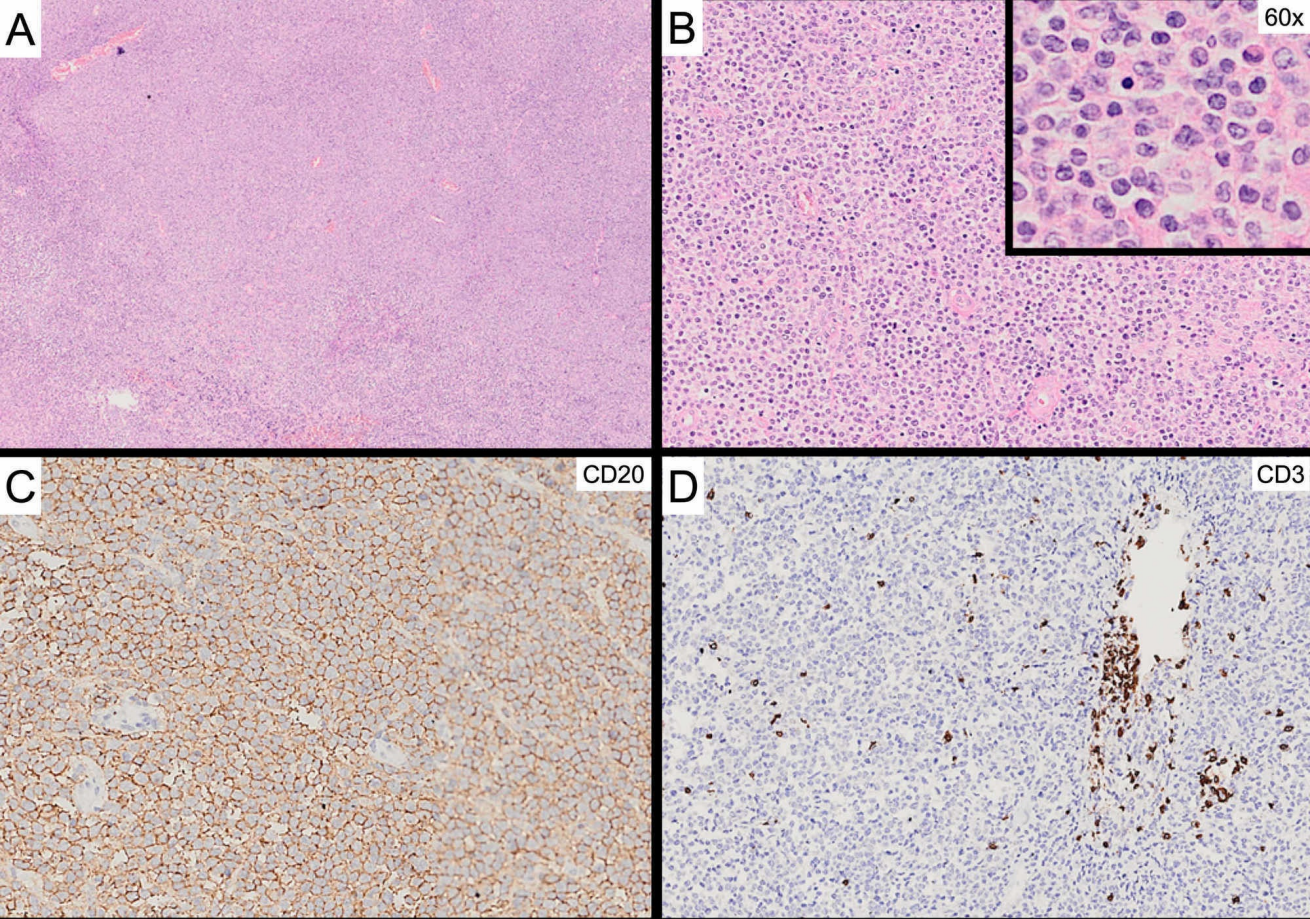
Pathology findings of the dural mass (A) Hematoxylin and eosin staining at 4x magnification of the dural lesion shows diffuse lymphoid infiltration; (B) 20x and 60x magnification shows oval cells with prominent large nucleoli; (C) Positive immunohistochemistry staining for CD20 in large cells; (D) Positive CD3 staining in mature lymphocytes consistent with diffuse large B-cell lymphoma.

Treatment with levetiracetam, midazolam, and dexamethasone for brain edema was started. Under the suspicion of meningioma, she underwent surgical resection of the dural mass without any complications; also colonoscopic biopsies of the appendicular mass were taken. Histologic analysis of both masses showed diffuse infiltration of large lymphoid cells; in immunohistochemistry, the large lymphoid cells were positive for cluster of differentiation 20 (CD20), B-cell leukemia/lymphoma 2 (BCL-2), BCL-6, and multiple myeloma oncogene 1 (MUM 1); and negative for CD3, CD10, and C-MYC, findings consistent with non-germinal center DLBCL (Figure 2). During the in-hospital stay, she remained seizure-free and died 17 days after admission due to ventilator-associated pneumonia and septic shock.

## Discussion

Secondary CNS lymphoma may present as a dural-based lesion mimicking meningiomas [4]. On MRI, lymphomas are typically isointense to hypointense relative to grey matter in the T2-weighted imaging sequence with intense postcontrast enhancement. Meningiomas are the most common intracranial tumors accounting for up to 30% of non-glial tumors of the CNS. As in this patient, they appear as a lobular, extra-axial mass with well-circumscribed margins mostly located in the parasagittal aspect of the cerebral convexity, the lateral hemisphere convexity, the sphenoid wing, middle cranial fossa, and the olfactory groove. On MRI, a meningioma is typically isointense to hypointense relative to grey matter in the T1-weighted sequence and isointense to slight hyperintense relative to grey matter on the T2-weighted sequence with avid homogeneous enhancement after contrast administration; they may occasionally have areas of central necrosis or calcification without gadolinium enhancement [5]. A thin linear enhancement along the dura infiltrating away from the lesion, known as the dural tail sign, was once thought to be a pathognomonic feature of meningiomas. This non-specific radiological sign may be found in up to 70% of the cases and 16% of meningioma mimics [5-6]. Several benign and malignant pathologies apart from lymphoma may also mimic some of these features. Among them are meningeal metastasis from solid tumors, solitary fibrous tumor, sarcoidosis, Erdheim-Chester disease, and Rosai-Dorfman disease [4]. We utilized an already established five key imaging features to aid in distinguishing between meningiomas from their mimics, such as our secondary DLBCL, which are as follows: a marked T2 hypointensity, marked T2 hyperintensity, osseous destruction, leptomeningeal extension, and lack of dural tail sign. Reports have indicated that two or more of these determining factors should be red flags for the clinician [6].

## Conclusions

When approaching a patient with a suspected meningioma, close attention to imaging features may help distinguish them from meningioma-like lesions. The distinction between meningioma and meningioma-like lesions is essential due to distinct changes in therapy depending on tissue-based pathology.
